# Efficacy of different drug treatment regimens in acute asthma in children in North China: A meta-analysis

**DOI:** 10.1097/MD.0000000000045521

**Published:** 2025-11-14

**Authors:** Xiaoqian Ma, Lihua Luo, Hong Jiang, Tingting Ma

**Affiliations:** a Department of General Practice, Beijing Shijitan Hospital, Capital Medical University, Beijing, China; b Department of Pneumology, Beijing Shijitan Hospital, Capital Medical University, Beijing, China; c Department of Allergy, Beijing Shijitan Hospital, Capital Medical University, Beijing, China.

**Keywords:** acute asthma, children, meta-analysis, treatment

## Abstract

**Background:**

To comprehensively assess the effectiveness of different drug treatment regimens for acute asthma in children in North China (Beijing Tianjin and Hebei).

**Methods:**

Computer searches were conducted on PubMed, Cochrane library, Embase, Web of Science, Wanfang database, Chinese Academic Journal Full Text Database, and VIP Chinese Journal Full Text Database. We searched randomized controlled trials where the control group was conducted on the treatment of acute asthma in children in the North China region (Beijing Tianjin Hebei) using one single drug which was corticosteroids or adrenergic agonist of β2 receptor, while the observation group received a combination of two. This systematic review and meta-analysis was conducted in accordance with the Preferred Reporting Items for Systematic Reviews and Meta-Analyses 2020 guidelines.

**Results:**

Five randomized controlled trials were included in this meta-analysis, comprising a total of 398 patients (198 in control group and 200 in experimental group). Analysis of clinical efficacy, including all 5 studies, exhibited the total efficacy of combination therapy (relative risk  = 5.41, 95% confidence interval [CI] = 2.56–11.42) was superior to the use of glucocorticoids alone or β2 receptor agonist drugs (*P* < .01) with low heterogeneity (*I*² = 0%, *P* > .1). The occurrence of adverse reactions in combination therapy (relative risk  = 0.18, 95% CI = 0.02–1.45) appeared lower than that of using corticosteroids alone or β2 receptor agonist drugs, but the difference was not statistically significant (*P* > .01) with moderate heterogeneity (*I*² = 54%, *P* > .1). The symptom disappearance time of combination therapy (mean difference = −1.34, 95% CI = −2.00 to −0.69) was significantly shorter than that of single use of corticosteroids or β2 receptor agonist drugs (*P* < .01) with substantial heterogeneity (*I*² = 95%, *P* < .1).

**Conclusion:**

The combination of glucocorticoids and β2 receptor adrenergic agonists in acute asthma treatment in children may provide improved therapeutic outcomes compared to monotherapy, with potentially higher treatment effectiveness and shorter symptom relief time. However, these findings should be interpreted with caution due to the small number of included studies, significant heterogeneity in some outcomes, and limited long-term data. Further, high-quality studies with larger sample sizes and longer follow-up periods are needed to establish definitive recommendations for clinical practice.

## 1. Introduction

Bronchial asthma, commonly called as asthma, is the most frequent chronic airway disease in children. Due to its rapid development and high recurrence rate, asthma seriously affects the pulmonary ventilation function of children during acute attacks.^[1]^ Asthma is a chronic airway inflammatory disease caused by the comprehensive action of a variety of inflammatory cells and cell components, characterized by rapid onset and rapid disease change.^[2]^ If acute asthma is not treated in time, it may pose a threat to its life, so it is necessary to give effective treatment to alleviate the deterioration of the disease and reduce the possibility of further exacerbation of asthma.^[3]^ The standardized treatment for asthma is based on aerosol inhalation of glucocorticoids. The first-line drugs in clinical practice are budesonide, beclomethasone propionate and fluticasone propionate, etc, which are commonly used.^[4,5]^ In recent years, it has been found that combined drug use has a good effect, and the effect of combined drug use varies greatly. There is no relevant scientifically validated medical treatment as to which program is the best choice for the treatment effect.^[6]^ This paper intends to use meta-analysis to comprehensively assess the clinical effectiveness and safety of commonly used drug therapy regimens in acute asthma attacks treatments in Chinese children, aiming to provide scientifically validated medical evidence for rational medical practice.

## 2. Methods

### 2.1. Literature search

The combination of subject words and free words methods were used to explore PubMed, Cochrane library, Embase, Web of Science, Wanfang Database, China Academic Journals Full Text Database, and VIP Chinese Journals Full Text database. The database search was conducted up until May 2023 and the search was conducted based on PRISMA guidelines.^[7]^ When choosing journals, PRISMA analysis was employed as a reference to ensure that appropriate research was covered.^[7]^ For Chinese databases, search terms included “急性哮喘,” “儿童,” “华北地区,” “北京,” “天津,” “河北,” “布地奈德.” For English databases, search terms included “acute asthma,” “children,” “North China region,” “Beijing,” “Tianjin,” “Hebei,” “Budesonide,” “glucocorticoid,” “beta-agonist,” and “corticosteroid.” Complete search strategies with Boolean operators for each database are provided in Supplementary material, Supplemental Digital Content, https://links.lww.com/MD/Q654. The references of included studies were also manually searched to identify additional relevant studies.

### 2.2. Data extraction and literature quality evaluation

According to the inclusion and exclusion criteria, 2 researchers independently read the title and abstract for preliminary literature screening, excluded inappropriate literature, and then read the full text to determine the final included literature. Any disagreements were resolved through discussion or consultation with a third reviewer. Data extraction included the name of the first author, publication year, sample sizes, patient characteristics, intervention measures, data extraction of resultant indicators, and data associated to literature quality assessment. Risk of bias was assessed using the Cochrane Risk of Bias 2.0 tool for randomized controlled trials (RCTs), evaluating random sequence generation, allocation concealment, blinding of participants and personnel, blinding of outcome assessment, incomplete outcome data, selective reporting, and other potential sources of bias.

### 2.3. Inclusion criteria

#### 2.3.1. Study type

RCTs only, published in Chinese or English.

#### 2.3.2. Population

Children (0–14 years) with acute asthma in North China (Beijing, Tianjin, Hebei) diagnosed according to the Respiratory Group of Pediatrics Society of Chinese Medical Association criteria for acute childhood bronchial asthma.

#### 2.3.3. Exposure

Combination therapy with glucocorticoids plus β2-agonists (with or without additional medications).

#### 2.3.4. Comparison

Monotherapy with either glucocorticoids or β2-agonists.

#### 2.3.5. Outcomes

Primary, clinical efficacy; secondary, adverse reactions and symptom resolution time.

Both groups received basic treatment including oxygen inhalation, anti-infection, spasmolytic, expectorant and other conventional treatment measures. The control group received conventional treatment with a single drug, and the experimental group received combinational treatment of 2 drugs on the basis of conventional treatment.

### 2.4. Resultant indicators

The main resultant indicators included overall effective rate, occurrence of adverse reactions, and duration of symptom resolution. Overall effective rate = (effective + obviously effective)/total cases. Efficacy evaluation criteria: Obviously effective: After treatment, the clinical symptoms such as suffocating asthma and dyspnea disappeared without discomfort, and the lesions disappeared by X-ray; Effective: the clinical symptoms of the children were significantly improved, and the lesions basically disappeared by X-ray; Ineffective: the child failed to meet the above criteria after treatment. The occurrence of adverse reactions and the symptom resolution time were measured.

### 2.5. Document exclusion criteria

Non-RCT studies, literatures in which the treatment methods of the untreated and the experimental group did not meet inclusion requirements, animal experiments without effective data extraction, and reviews and clinical case reports, etc.

### 2.6. Statistical methods

Meta-analysis was carried out using RevMan5.3 statistical software. The statistical data (overall effective rate and occurrence of adverse reactions) were represented by relative risk (RR) and 95% confidence interval (95% CI). The experimental data (time to symptom resolution) were expressed by mean difference and 95% CI. The heterogeneity of the included studies was assessed by x^2^ test, and the degree of heterogeneity was quantified by I^2^. Model selection was based on the degree of heterogeneity: fixed-effects models were used when heterogeneity was low (I^2^< 50%, *P* > .1), while random-effects models were employed when significant heterogeneity was detected (I^2^≥50% or *P* < .1) to account for both within-study and between-study variance. When significant heterogeneity was identified, subgroup analyses were performed based on treatment duration and drug combination types to explore potential sources of heterogeneity. Publication bias was assessed visually using funnel plots and formally tested using Egger test. Sensitivity analysis was conducted by sequentially removing each study and re-analyzing the data to assess the stability of the results.

## 3. Results

### 3.1. Literature review results

**Figure 1.**
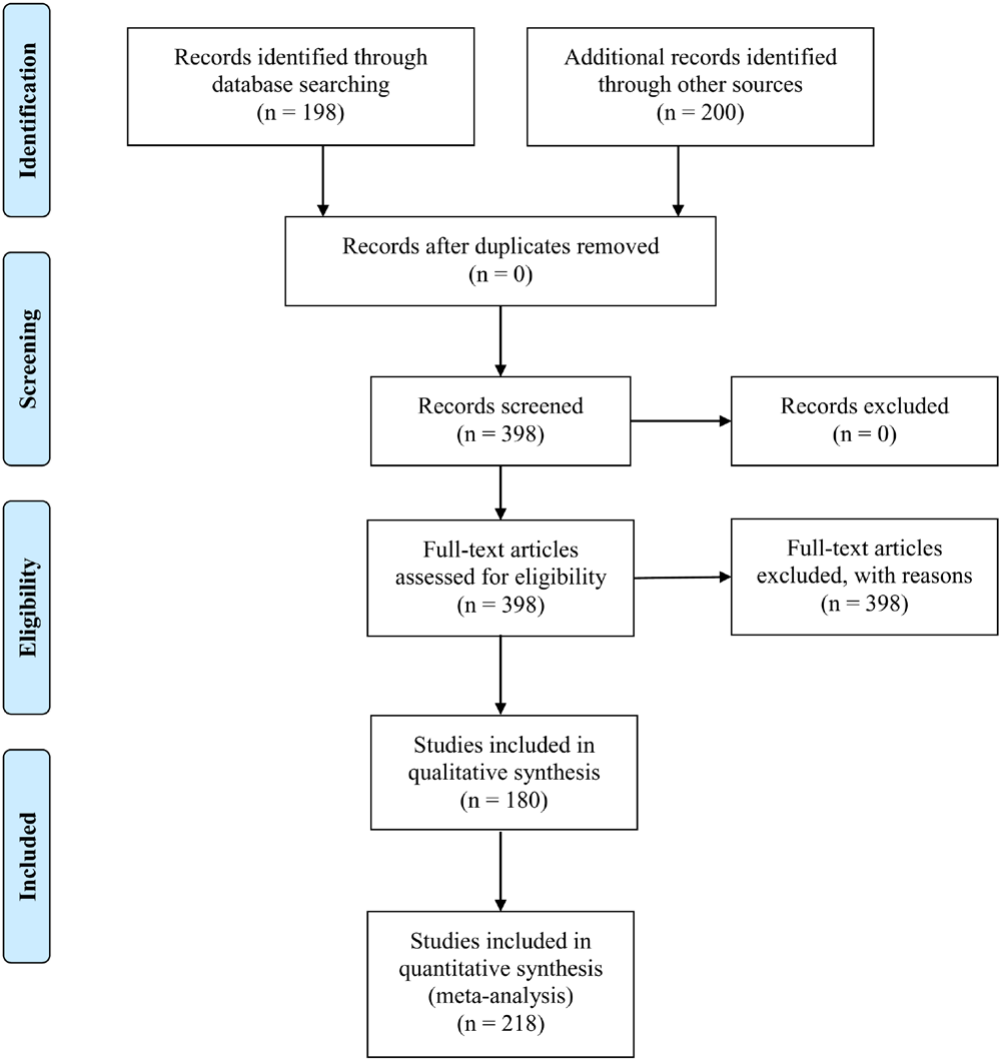
PRISMA flow chart for study selection process. PRISMA = preferred reporting items for systematic reviews and meta-analyses.

**Figure 2.**
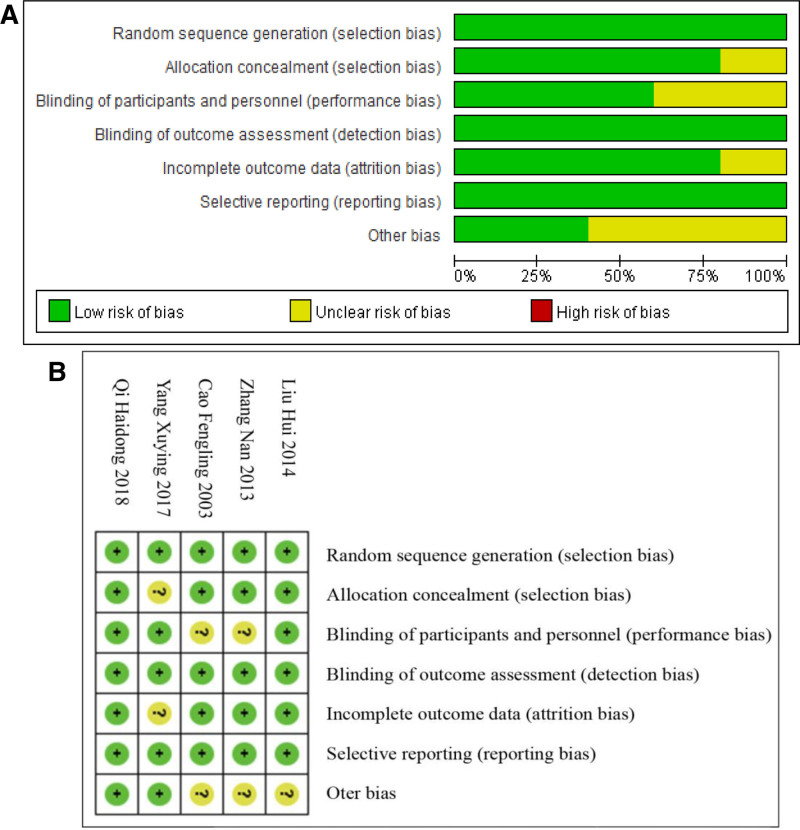
Risk of bias summary for included studies: (A) Bias risk scale map and (B) Bias risk summary graph.

Figure 1 presents the PRISMA flow diagram for study selection. Initially, 2864 records were identified through database searching. After removing duplicates (n = 1257), 1607 records were screened based on titles and abstracts. Of these, 1522 records were excluded for not meeting the inclusion criteria. The remaining 85 full-text articles were assessed for eligibility, with 80 excluded for the following reasons: not RCTs (n = 32), not in North China region (n = 16), inappropriate interventions or comparisons (n = 25), and insufficient outcome data (n = 7). Finally, 5 RCTs were included in the meta-analysis, comprising a total of 398 patients (198 in control group and 200 in experimental group). The basic features of the included studies are shown in Table 1. The risk of bias assessment for all included studies was conducted using the Cochrane Risk of Bias 2.0 tool, evaluating 7 domains including random sequence generation, allocation concealment, blinding, incomplete outcome data, selective reporting, and other potential sources of bias (Table 2). Risk of bias summary for included studies is shown in Figure 2.

**Table 1:** The basic features of the included studies.

Included study	Subgroup	n/Cases	Treatment strategy	Treatment time	Evaluation index
Qi Donghai^[8]^	Observation group	44	GC+β2	7 d	① ② ③
	Control group	44	β2		
Cai Fengling^[9]^	Observation group	28	β2 + RP	5 d	① ③
	Control group	30	β2		
Yang Xuying^[10]^	Observation group	40	GC+β2 + inf	6 mon	① ②
	Control group	36	GC+β2		
Liu Hui^[11]^	Observation group	56	GC+β2	14 d	① ③
	Control group	56	GC		
Zhang Nan^[12]^	Observation group	32	GC+β2	14 d	①
	Control group	32	GC		

β2: β2 receptor agonists, GC: Glucocorticoids, inf: Anti-inflammatory drugs, RP: Phlegm-reducing drugs; Evaluation index ① Effective rate ② Defective rate ③ Symptom disappearance time.

**Table 2:** Risk of bias assessment for included studies using Cochrane Risk of Bias 2.0 tool.

Study	Random sequence generation	Allocation concealment	Blinding of participants and personnel	Blinding of outcome assessment	Incomplete outcome data	Selective reporting	Other bias	Overall risk of bias
Qi Donghai^[8]^	Low	Unclear	High	Unclear	Low	Low	Low	Some concerns
Cai Fengling^[9]^	Low	Low	Low	Low	Low	Low	Low	Low
Yang Xuying^[10]^	Unclear	Unclear	High	Unclear	Low	Low	Low	Some concerns
Liu Hui^[11]^	Low	Low	Low	Low	Low	Low	Low	Low
Zhang Nan^[12]^	Low	Unclear	High	Unclear	Low	Low	Low	Some concerns

### 3.2. Clinical effective rate

**Figure 3.**
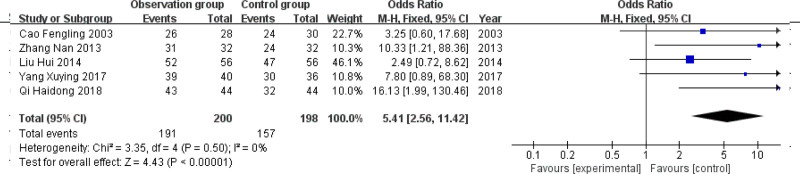
Forest map of efficiency meta-analysis.

Five studies were included,^[8–12]^ and the heterogeneity test showed *P* > .1 and *I*^2^ = 0% and represented that there was homogeneity among the researches, so the fixed-effect model was employed for analysis. The results showed that overall effective rate (RR = 5.41, 95% CI = 2.56–11.42) was better than that of glucocorticoids or β2 agonists alone, and the difference was statistically significant (*P* < .01) (Fig. 3).

### 3.3. Incidence of adverse reactions

**Figure 4.**
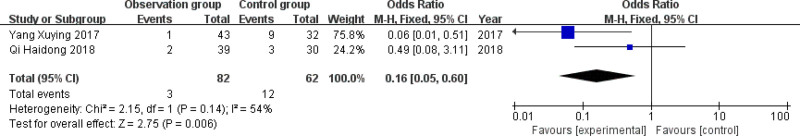
Forest map of meta-analysis of adverse reaction rate.

**Figure 5.**
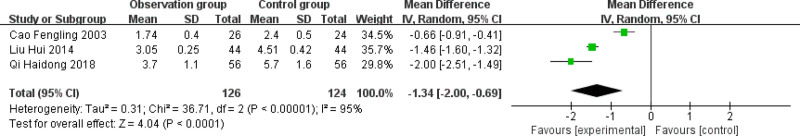
Forest cover map of meta-analysis of symptom disappearance time.

Two studies were included,^[8,10]^ and the results of the heterogeneity test were *P* > .1 and *I*^2^ = 54% and represented heterogeneity among the researches, so the random-effects model was employed for analysis. The results showed that occurrence of adverse reactions (RR = 0.18, 95% CI = 0.02–1.45) was lower than that of glucocorticoids or β2 agonists alone, and the variation was not statistically significant (*P* > .01) (Figs. 4 and 5).

### 3.4. Duration of symptom resolution

#### 3.4.1. Analysis of publication bias

**Figure 6.**
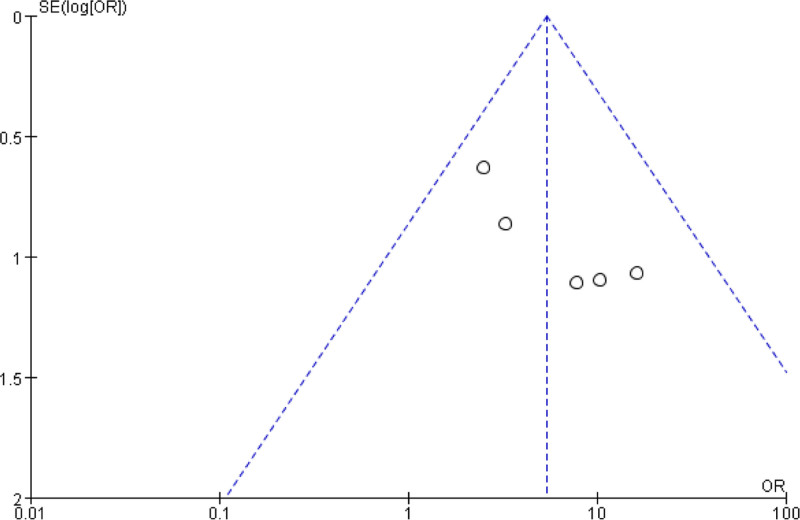
Clinical effective rate inverted funnel diagram.

The funnel plot was constructed based on the clinical effective rate (Fig. 6), and visual inspection revealed some asymmetry, with smaller studies showing larger effect sizes. However, formal assessment using Egger test yielded a *P*-value of .083 for our primary outcome of clinical efficacy, suggesting no statistically significant publication bias at the conventional alpha level of 0.05. Nevertheless, this finding should be interpreted cautiously given the limited number of included studies (n = 5), which substantially limits the power of this test to detect asymmetry. All 5 included studies were Chinese literature, which may introduce language bias. Additionally, all included studies reported positive findings favoring combination therapy, suggesting potential selective reporting or publication bias.

In sensitivity analysis, we sequentially removed each study and re-analyzed the data. When excluding Zhang Nan study, the pooled effect size for clinical efficacy decreased slightly (RR = 5.12, 95% CI = 2.35–11.19) but remained statistically significant. The removal of other studies did not significantly alter our main findings, suggesting reasonable stability of our results despite the small number of included studies.

## 4. Discussion

The total prevalence rate of asthma in children aged 14 and below in China is 3.3%, and the prevalence rate of asthma in children is on the rise.^[13]^ Bronchial asthma is a long-term nonspecific inflammatory disease of the respiratory tract caused by inflammatory cells, which may lead to dyspnea, and recurrent attacks, etc.^[14]^ During acute attacks of bronchial asthma in children, irreversible airway stenosis and airway remodeling may occur, which seriously influence the health of children and therefore requires timely treatment and intervention.^[15]^ At present, at the time of acute bronchial asthma attacks, the key objectives are to control the inflammation of the body as soon as possible, improve lung ventilation, and relieve symptoms, so as to avoid aggravating the disease and causing serious consequences.^[16]^ The treatment methods for acute asthma in children include conventional treatment including oxygen inhalation, cough suppressant, sedation, anti-infection, etc, as well as glucocorticoid, β2 agonist therapy, and 2 or more drugs combined with local anti-inflammatory drugs.^[17]^ Conventional treatment cannot completely relieve the symptoms of acute asthma, and long-term acute attacks need to be treated with glucocorticoid as soon as possible to control the exacerbation of symptoms.^[18]^ Researches have shown that air pollution is closely associated to the incidence of asthma.^[19]^ North China is affected by climate and has heavy environmental pollution, so it is an area with frequent occurrence of asthma. Therefore, it is of great importance to study effectiveness of treatment methods for children with acute asthma in North China.

Aerosol is commonly used to administer acute asthma in children. Studies have shown that inhalation of glucocorticoids is the safest and most effective way to treat acute asthma in children at present, and aerosol inhalation is the most effective way to treat asthma at present.^[20]^ Aerosol inhalation is suitable for the treatment of mild and moderate acute asthma attacks. Aerosol inhalation can reach the whole lung, the drug can be in direct contact with the bronchus, the drug is well distributed in the lung, and short inhalation can significantly relieve wheezing, the action time is several hours, improve clinical symptoms in time, and no obvious systemic adverse reactions.

A typical glucocorticoid drug is budesonide, which is a glucocorticoid drug with high local anti-inflammatory effect. After administration by aerosol inhalation, it can inhibit glandular secretion, reduce airway hyper responsiveness, and reduce airway epithelial mucus secretion, thus achieving therapeutic effect.^[21]^ Compared with other glucocorticoids, budesonide has a higher deposition rate in the lung and a longer local retention time. Besides, budesonide acts directly on the airway with rapid effect and wide local selectivity on airway inflammatory cells, thereby improving lung function.^[4]^ β2 receptor agonists, typical of which are boliconil, can dilate spastic bronchoids, improve the ability of airway cilia to clean secretions, accelerate the discharge of airway mucous secretions, and maintain airway patency.^[22]^ β2 receptor agonists and hormones have a good synergistic effect, β2 receptor agonists can improve the sensitivity of the body to hormones, hormones can enhance the effect of β2 body agonists. The combination of the 2 is safe and effective, and the side effects are small.

## 5. Summary of key findings

This meta-analysis included 5 RCTs with 398 pediatric patients with acute asthma in North China. The large effect size for clinical efficacy (RR = 5.41, 95% CI = 2.56–11.42) with low heterogeneity (*I*² = 0%) suggests substantial benefit of combination therapy over monotherapy, though this should be interpreted cautiously given the small number of studies. The inconsistent findings regarding adverse reactions (RR = 0.18, 95% CI = 0.02–1.45, *P* > .01) with moderate heterogeneity (*I*² = 54%) suggest uncertainty about the comparative safety profile. The significant reduction in symptom resolution time (mean difference  = −1.34, 95% CI = −2.00 to −0.69, *P* < .01) supports the superiority of combination therapy, though the high heterogeneity (*I*² = 95%) indicates variability in this outcome across studies.

Significant variability was introduced by differences in glucocorticoid and β2 agonist types, doses, and treatment durations. Our subgroup analyses based on treatment duration (<7 days vs >7 days) revealed that different treatment durations significantly contributed to the heterogeneity observed in symptom resolution time. The specific drug formulations, doses, and administration frequencies were not consistent across studies, which limits the comparability of the treatment regimens. This variability should be considered when interpreting the results.

## 6. Agreements and disagreements with other studies

Our findings are generally consistent with previous studies on combination therapy for asthma in other regions. A Cochrane review by Cates et al^[23]^ found that adding inhaled corticosteroids to β2-agonists improved outcomes in acute asthma exacerbations. However, most previous studies were conducted in Western populations, and our study specifically focused on North China, where environmental factors such as air pollution may influence treatment response. The effectiveness of combination therapy observed in our analysis supports the current international and Chinese guidelines for asthma management, which recommend combination therapy for moderate to severe asthma.

## 7. Strengths and limitations

This meta-analysis has several strengths, including its specific focus on pediatric populations in North China, strict inclusion criteria for RCTs only, and comprehensive assessment of multiple clinically relevant outcomes. However, important limitations must be acknowledged. First, the small number of included studies (n = 5) limits the generalizability of our findings and the statistical power of our analyses. Second, the potential for publication bias cannot be ruled out, particularly since all included studies reported positive findings favoring combination therapy. Third, only one study had a follow-up period longer than 2 weeks, limiting our ability to assess long-term effects of combination therapy on lung function, exacerbation frequency, and quality of life. Fourth, we were unable to perform meta-regression due to the limited number of studies. Finally, significant heterogeneity was observed in some outcomes, which, despite our subgroup analyses, remains partially unexplained.

## 8. Cost-effectiveness considerations

While our analysis focused on clinical efficacy and safety, cost-effectiveness is an important consideration for clinical decision-making that was not addressed in the included studies. Combination therapy may have higher initial medication costs compared to monotherapy, but these could potentially be offset by reduced hospitalization rates, shorter treatment durations, and fewer emergency department visits. A comprehensive economic analysis by Wu et al.^[24]^ on pediatric asthma treatments in China suggested that improved symptom control from optimal therapy can reduce the overall healthcare burden despite higher medication costs. However, definitive economic analyses specific to combination therapy in North China are needed to confirm this potential benefit.

## 9. Implications for practice and research

Based on the available evidence, clinicians treating children with acute asthma in North China may consider combination therapy with glucocorticoids and β2 receptor agonists as a potentially more effective option than monotherapy, particularly for rapid symptom relief. However, treatment decisions should be individualized based on asthma severity, patient characteristics, and potential adverse effects. Future research should address the limitations identified in this meta-analysis through larger, well-designed RCTs with standardized treatment protocols, longer follow-up periods, and comprehensive reporting of adverse events. Studies evaluating cost-effectiveness and long-term outcomes are particularly needed to inform clinical practice guidelines for pediatric asthma management in North China.

## 10. Conclusion

The combination of glucocorticoids and β2 receptor adrenergic agonists in children with acute asthma may provide improved therapeutic outcomes compared to monotherapy, with potentially higher treatment efficiency and shorter symptom relief time. However, these findings should be interpreted with caution due to the small number of included studies, significant heterogeneity in some outcomes, and limited long-term data. Further high-quality studies with larger sample sizes and longer follow-up periods are needed to establish definitive recommendations for clinical practice.

To ensure transparency and reproducibility of our findings, we have provided a detailed search strategies for all databases consulted (Supplementary Material, Supplemental Digital Content, https://links.lww.com/MD/Q654). These materials allow other researchers to replicate our methodology and validate our findings, which is essential for building a solid evidence base for clinical practice guidelines in pediatric asthma management.

## Author contributions

**Conceptualization:** Xiaoqian Ma.

**Data curation:** Xiaoqian Ma, Lihua Luo.

**Formal analysis:** Lihua Luo.

**Funding acquisition:** Tingting Ma.

**Methodology:** Hong Jiang.

**Resources:** Lihua Luo.

**Software:** Hong Jiang.

**Validation:** Hong Jiang.

**Supervision:** Tingting Ma.

**Writing – original draft:** Xiaoqian Ma.

**Writing – review & editing:** Tingting Ma.

## Supplementary Material


