# Supplementary material for: Efficacy of different drug treatment regimens in acute asthma in children in North China: A meta-analysis
Source: Medicine (Baltimore). 2025 Nov 14;104(46):e45521. doi: 10.1097/MD.0000000000045521 (PMC12622650; doi:10.1097/MD.0000000000045521)
Supplement: Supplementary file 1 [file medi-104-e45521-s001.pdf]

Supplementary Material Detailed Search Strategies

1. PubMed

```
(asthma[MeSH] OR asthma*[tiab] OR "bronchial asthma"[tiab])
AND
(child*[tiab] OR pediatric*[tiab] OR paediatric*[tiab] OR infant*[tiab])
AND
("North China"[tiab] OR Beijing[tiab] OR Tianjin[tiab] OR Hebei[tiab])
AND
(glucocorticoid*[tiab] OR corticosteroid*[tiab] OR budesonide[tiab] OR "beta-agonist*"[tiab] OR "beta 2 agonist*"[tiab] OR salbutamol[tiab] OR terbutaline[tiab] OR "combination therapy"[tiab])
```

2. Cochrane Library

```
#1 MeSH descriptor: [Asthma] explode all trees
#2 asthma*:ti,ab,kw
#3 "bronchial asthma":ti,ab,kw
#4 #1 OR #2 OR #3
#5 child*:ti,ab,kw
#6 pediatric*:ti,ab,kw OR paediatric*:ti,ab,kw
#7 infant*:ti,ab,kw
#8 #5 OR #6 OR #7
#9 "North China":ti,ab,kw
#10 Beijing:ti,ab,kw
#11 Tianjin:ti,ab,kw
#12 Hebei:ti,ab,kw
#13 #9 OR #10 OR #11 OR #12
#14 glucocorticoid*:ti,ab,kw
#15 corticosteroid*:ti,ab,kw
#16 budesonide:ti,ab,kw
#17 "beta-agonist*":ti,ab,kw
#18 "beta 2 agonist*":ti,ab,kw
#19 salbutamol:ti,ab,kw
#20 terbutaline:ti,ab,kw
#21 "combination therapy":ti,ab,kw
#22 #14 OR #15 OR #16 OR #17 OR #18 OR #19 OR #20 OR #21
#23 #4 AND #8 AND #13 AND #22
```

3. Embase

```
('asthma'/exp OR asthma*:ti,ab OR 'bronchial asthma':ti,ab)
AND
(child*:ti,ab OR pediatric*:ti,ab OR paediatric*:ti,ab OR infant*:ti,ab)
AND
('north china':ti,ab OR beijing:ti,ab OR tianjin:ti,ab OR hebei:ti,ab)
AND
(glucocorticoid*:ti,ab OR corticosteroid*:ti,ab OR budesonide:ti,ab OR 'beta-agonist*':ti,ab
OR 'beta 2 agonist*':ti,ab OR salbutamol:ti,ab OR terbutaline:ti,ab OR 'combination
therapy':ti,ab)
AND
[embase]/lim
```

4. Web of Science

```
TS=((asthma* OR "bronchial asthma")
AND
(child* OR pediatric* OR paediatric* OR infant*)
AND
("North China" OR Beijing OR Tianjin OR Hebei)
AND
(glucocorticoid* OR corticosteroid* OR budesonide OR "beta-agonist*" OR "beta 2 agonist*" OR
salbutamol OR terbutaline OR "combination therapy"))
```

5. CNKI (Chinese Database)

```
KY=('哮喘' OR '支气管哮喘')
AND
KY=('儿童' OR '小儿' OR '婴幼儿' OR '儿科')
AND
KY=('华北' OR '北京' OR '天津' OR '河北')
AND
KY=('糖皮质激素' OR '皮质类固醇' OR '布地奈德' OR 'β2受体激动剂' OR '沙丁胺醇' OR '特布他林' OR
'联合治疗')
```

6. Wanfang Database (Chinese Database)

```
主题:('哮喘' OR '支气管哮喘')
AND
主题:('儿童' OR '小儿' OR '婴幼儿' OR '儿科')
AND
主题:('华北' OR '北京' OR '天津' OR '河北')
AND
主题:('糖皮质激素' OR '皮质类固醇' OR '布地奈德' OR 'β2受体激动剂' OR '沙丁胺醇' OR '特布他林' OR '联合治疗')
```

7. VIP Database (Chinese Database)

```
主题:('哮喘' OR '支气管哮喘')
AND
主题:('儿童' OR '小儿' OR '婴幼儿' OR '儿科')
AND
主题:('华北' OR '北京' OR '天津' OR '河北')
AND
主题:('糖皮质激素' OR '皮质类固醇' OR '布地奈德' OR 'β2受体激动剂' OR '沙丁胺醇' OR '特布他林' OR '联合治疗')
```

Notes:

- Search date range: Inception to May 2023
- No language restrictions were applied in the database search, but only English and Chinese publications were included as per inclusion criteria
- MeSH terms were used where applicable
- Title, abstract, and keyword fields were searched for most terms
- Additional manual searches of reference lists from included studies were conducted
